# Genetics of ovulatory dysfunction and infertility: a scoping review and gene ontology analysis

**DOI:** 10.3389/fendo.2025.1458711

**Published:** 2025-06-04

**Authors:** Erin E. DiPietro, Sara M. Sarasua, Casey S. Hopkins, Satishkumar Ranganathan Ganakammal, Luigi Boccuto, Joshua Hurwitz

**Affiliations:** ^1^ School of Nursing, Clemson University, Clemson, SC, United States; ^2^ Illume Fertility Center, Norwalk, CT, United States

**Keywords:** ovulatory and anovulatory cycles, ovulatory dysfunction, genetics, infertility, ovulatory dysfunctional infertility, genes

## Abstract

**Background:**

The genetic components of the etiologies of ovulatory dysfunction-related infertility (ODRI) are poorly characterized.

**Objectives:**

This paper aimed to comprehensively identify, compile, and categorize published research on relationships between genetics and ovulatory-related infertility in humans.

**Methods:**

A scoping review was performed on research articles relating human genes, ovulatory dysfunction, and infertility retrieved from PubMed and Web of Science databases. A total of 45 articles were included in the study. The data has been organized into three categories based on relevant findings: polycystic ovary syndrome (PCOS), premature ovarian insufficiency (POI), and other diagnoses related to ovulatory dysfunction and infertility.

**Results:**

Sources revealed 235 different genes linked to ovulatory dysfunction and infertility including follicle-stimulating hormone receptor (*FSHR*), luteinizing hormone/choriogonadotropin receptor (*LHCGR*), and bone morphogenic protein 15 (*BMP15*). PCOS-related articles revealed variants in genes with functions focused on androgen production, such as *LHCGR* and *FSHR*. POI-related articles revealed variants in genes with functions focused on folliculogenesis and pubertal development, such as *BMP15* and *STAG3*, stromal antigen 3. The “other” category revealed genes resulting in enzyme deficiencies interacting with a wide range of functions.

**Conclusions:**

In this review, we have highlighted the extreme variability in what is known about the genetics of ODRI by compiling and categorizing genes identified in the literature as associated with ODRI and its associated subtypes. We have also provided a comprehensive list of ODRI genes specifically identified in humans. The findings from this review, specifically the list of ODRI genes, can be used for targeted gene panel development in assisted reproductive technology to improve clinical testing and diagnosis, as well as in developing individualized treatment strategies for ODRI patients.

## Introduction

Fertility is the ability to achieve a clinical gestation, but the definition of infertility is not as clear (1). The exact constraints used to define infertility were previously convoluted; however, the American Society for Reproductive Medicine (ASRM) recently redefined the condition, as follows: Infertility can be identified in individuals who are unable to conceive, requiring medical intervention. The period of attempted conception before diagnosis of infertility and medical intervention is a standard of one year for female patients under the age of 35 and six months for female patients 35 years of age or older (2).

Ovulation dysfunction is one of the most common conditions associated with infertility (3). It can manifest in different ways. Anovulation is the complete absence of ovulation. Oligoovulation, or infrequent ovulation, is an inconsistent ovulation pattern that is usually paired with irregular menstruation patterns (4). Ovulatory dysfunction is observed in many different disorders and conditions, including the most common one, polycystic ovary syndrome (PCOS), affecting an estimated 10-13% of the female population around the world (5, 6).Certain cardinal components are used to initially diagnose PCOS, specifically, ovulatory dysfunction, clinical or biochemical hyperandrogenism, and polycystic ovarian morphology on a sonographic visualization (5). A positive diagnosis of PCOS is made if the patient has at least two of these components (6). PCOS is a complex disorder, affecting the body at the endocrine and metabolic levels (7, 8). At the endocrine and metabolic level, PCOS patients may suffer from insulin resistance and hyperandrogenism. At the phenotypic level, PCOS patients may suffer from comorbidities such as type two diabetes, hypertension, dyslipidemia, and obesity. It is imperative to highlight that in the classification of the disease, the clinical manifestations vary significantly (8). The variance in manifestations of PCOS has led to subcategorization. Type I classic PCOS is characterized by increased LH and LH/FSH ratio, increased androgens, elevated insulin levels, polycystic ovarian morphology, and increased waist measurements. Type II classic PCOS is characterized by all characteristics of Type I but without polycystic ovarian morphology. Ovulatory PCOS has milder hormonal imbalances and clinical implications. Normoandrogenic PCOS is the rarest form and has no clinical presentation except for a heightened LH and LH/FSH ratio (9).

There are some known gene associations that exist for PCOS. The primary category of genes with a known association with PCOS is involved with the endocrine system. These gene functions are involved with hormone genesis, hormone regulation, hormone action, and hormone secretion (5). Other gene associations to PCOS include the *Fat Mass Obesity* gene and the *PCOS1* gene. While PCOS accounts for an estimated 80% of ovulatory dysfunction-related infertility (ODRI) cases (3), a diagnosis of PCOS does not directly *cause* infertility (6).

Primary Ovarian Insufficiency (POI) is a condition leading to abnormal or complete lack of ovulation before the age of 40 (10, 11). To confirm a diagnosis of POI, a biologically female patient must be below the age of 40, have repeated elevated levels of Follicle Stimulating Hormone reported on two separate occasions at least four weeks apart, and have menstrual disturbance (11–13). POI is estimated to be present in approximately 1% of the female population (13).

It is known that chromosomal anomalies, such as structural translocations or deletion of an X chromosome, are associated with approximately 15% of POI cases (14), thus our focus is on patients without an obvious cytogenetic association. There are many genes associated with POI. The general functions of these genes include folliculogenesis, follicular development, and ovarian steroidogenesis (14). Identifying and clarifying these gene associations for POI will allow researchers and clinicians to better understand the underlying factors of the condition (11).

Other causes of ovulatory dysfunction and subsequent infertility are obesity, hormone-regulatory disorders, and hypothalamic amenorrhea. A small percentage of this population remains idiopathic, or unexplained (15). Some genes that have documented associations in this subgroup are involved with recombination, synapsis, and DNA mismatch repair (16).

In this scoping review, a systematic search of the literature was performed for genes involved with ovulatory dysfunction and infertility. The aim was to create a record of documented gene associations between ovulatory dysfunction and infertility, and to characterize how these genetic variants may result in female infertility. Secondarily, the aim was to generate a comprehensive list of ODRI-associated genes to serve as a foundation for future research efforts. The research question was: are there associations in the peer-reviewed literature between genetic variants and phenotypic ovulatory dysfunction in infertility patients?

## Methods

### Search strategy and article selection

**Figure 1:**
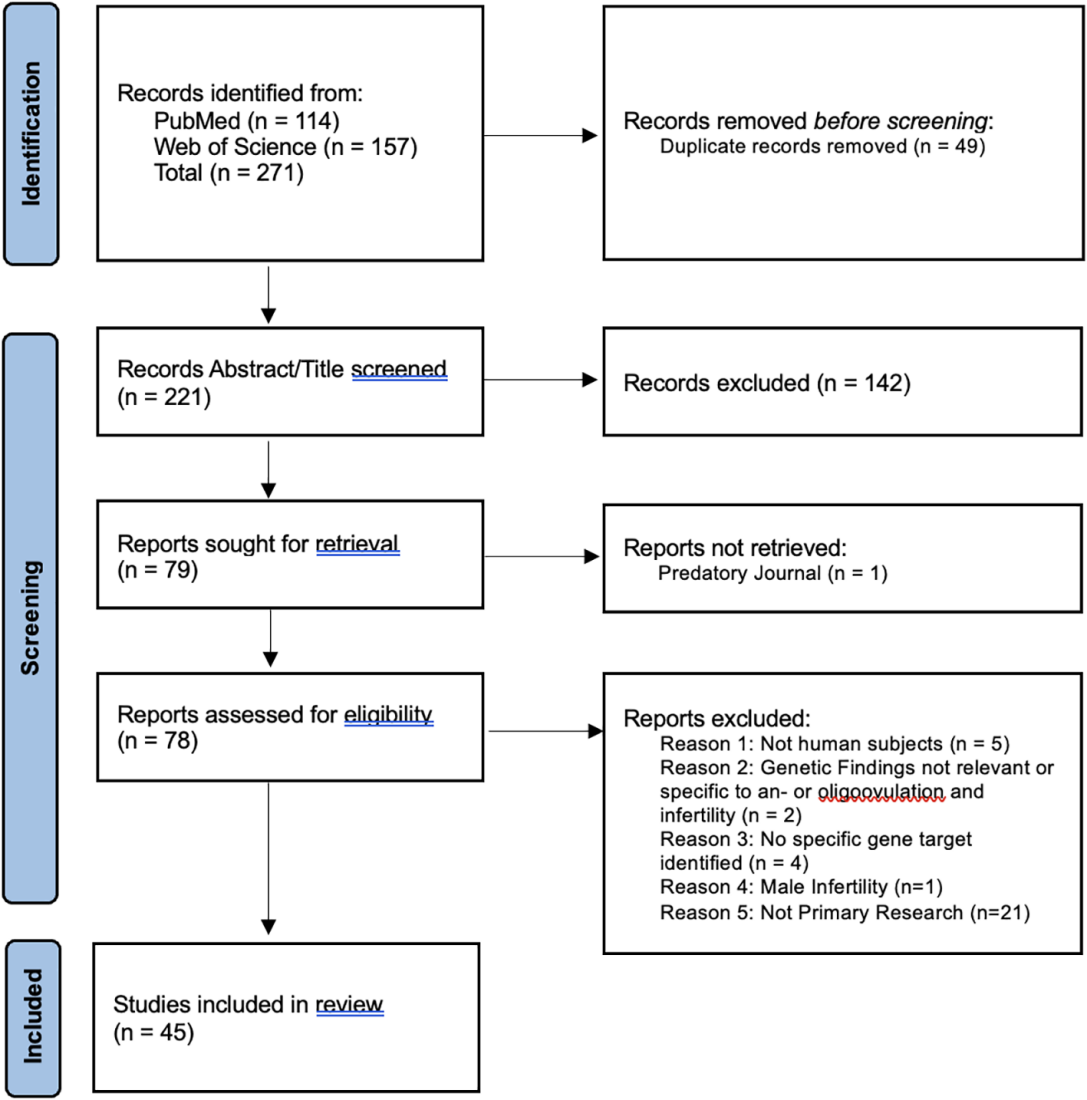
PRISMA 2020 flow diagram chart documenting article retrieval from PubMed and Web of Science and subsequent analysis for eligibility criteria. The primary reviewer participated in all steps, with a second and third reviewer validating the “Records Abstract/Title screened” step.

The PRISMA Guideline for Scoping reviews (17) was used to structure the search and analysis of literature included in the study (**Figure 1**). PubMed and Web of Science databases were queried for papers relevant to the genetics of ovulatory dysfunction and infertility. The search terms used for each database are included in **Table 1**. Key concepts used to build the queries were female infertility, genes, and ovulatory dysfunction. In addition to the key concepts, all Medical Subject Headings (MeSH) terms were used to increase the breadth of literature retrieved. A single reviewer was responsible for gathering, extracting, and analyzing data in a systematic fashion.

**Table 1:** Search queries designed for each database included in the literature search.

Database	Actual Query
PubMed	(((infertility[Title/Abstract] OR fertility[Title/Abstract]) AND (female[Title/Abstract] OR biologically female[Title/Abstract])) OR "Infertility, Female/genetics"[Mesh]) AND ((genes[Title/Abstract] OR genes[Title/Abstract] OR genetic[Title/Abstract] OR genetics[Title/Abstract] OR heredity[Title/Abstract] OR heritability[Title/Abstract]) OR "Genetics"[Mesh]) AND ((anovulation[Title/Abstract] OR anovulatory[Title/Abstract] OR amenorrhea[Title/Abstract] OR oligomenorrhea[Title/Abstract] OR menstrual disturbance[Title/Abstract]) OR "Anovulation"[Mesh]) NOT (animal OR animals)
Web of Science	(((TI=infertility OR AB=infertility) OR (TI=fertility OR AB=fertility)) AND ((TI=female OR AB=female) OR (TI="biologically female" OR AB="biologically female")))AND ((TI=genes OR AB=genes) OR (TI=genes OR AB=genes) OR (TI=genetic OR AB=genetic) OR (TI=genetics OR AB=genetics) OR (TI=heredity OR AB=heredity) OR (TI=heritability OR AB=heritability))AND ((TI=anovulation OR AB=anovulation) OR (TI=anovulatory OR AB=anovulatory) OR (TI=amenorrhea OR AB=amenorrhea) OR (TI=oligomenorrhea OR AB=oligomenorrhea) OR (TI="menstrual disturbance" OR AB="menstrual disturbance"))

Duplicate articles were eliminated using a duplicate removal tool in RefWorks (18). A secondary elimination of duplicates was performed manually due to the unique characters of foreign names from the two databases. After duplicate removal, a tab-delimited file containing general article citation information and abstracts was retrieved from RefWorks software.

Article titles and abstracts were reviewed for inclusion and exclusion criteria. Exclusion criteria included male infertility, whole chromosome abnormalities, epigenetic modulation, and chromosomal structural rearrangements. Inclusion criteria, aside from the key concepts, were human subjects, the English language, at least one genetic anomaly identified, and classification as a research article (e. g., not a review or editorial article). There was no start date restriction. The end date restriction was October 4^th^, 2023.

The primary reviewer was responsible for retrieving articles, duplicate removal, reviewing for inclusion and exclusion criteria, data extraction, and data analysis. A secondary review of the titles and abstracts was split between a second and third reviewer. If the information in the abstract was either insufficient or caused disagreement between the reviewers at the validation of inclusion and exclusion criteria, the paper was evaluated in its entirety by all three reviewers.

### Data extraction

A table was created to include the article title, primary author, year, and relevant categories of data. The type of study design and primary study location were extracted from each included article. The category for condition/disease type was broken down into PCOS, POI, or Other, with specific disease affiliation noted. Finally, the type of genetic testing/sequencing was recorded for each article. Any gene with a variant identified as affiliated with ODRI and its projected function was extracted from each article.

### Data synthesis and analysis

Gene names used for data inference and analysis were cross-checked using RefSeqGene (19). A complete list of gene names associated with ODRI was extracted from the research articles including the associated condition category, PCOS, POI, or Other. The ClinVar database (20) was queried for genes referenced in a higher number of research articles to identify if existing variants have been previously identified as pathogenic or likely pathogenic. The entire gene list was also used for a Gene Ontology (GO) analysis using the gprofiler2 (21) package in R to identify enriched functions of the gene names extracted from the research articles. The p-value cutoff used for GO-term enrichment analysis was p < 1e-16, adjusted for false positive discovery (21). The GO-term enrichment analysis was used to validate that the construct used in this review for categorization of ODRI was appropriate from a genetic stance.

## Results

### Article selection and inclusion

**Figure 1** illustrates how articles were obtained and included in this review. Articles retrieved from the PubMed and Web of Science databases contained 49 duplicates. After duplicate removal, 221 unique titles and abstracts were analyzed for inclusion and exclusion criteria. A total of 142 records were excluded after initial screening for eligibility. One article was not recovered due to predatory publishing.

Out of 78 retrieved articles, 33 did not meet the inclusion criteria upon further analysis. These articles were excluded due to the use of non-human subjects (n=5), genetic findings lacking specific relevance to ovulatory dysfunction and infertility (n=2), no specific gene variant(s) identified (n=4), structural rearrangements (n=4), publication type (n=17), and male infertility (n=1). A total of 45 articles were included in the full article review.

### Data extraction

Research article types retrieved and included in this review are case reports (n=20), observational studies (n=22), and experimental studies (n=3). They originate from 21 unique countries. Thirty-eight reports exclusively used DNA sequencing to observe variants in gene loci involved with ovulatory dysfunction. Two articles used transcriptome profiling exclusively and three used both DNA sequencing and RNA profiling to demonstrate how a mutated gene impacts ovulatory outcome.

**Figure 2:**
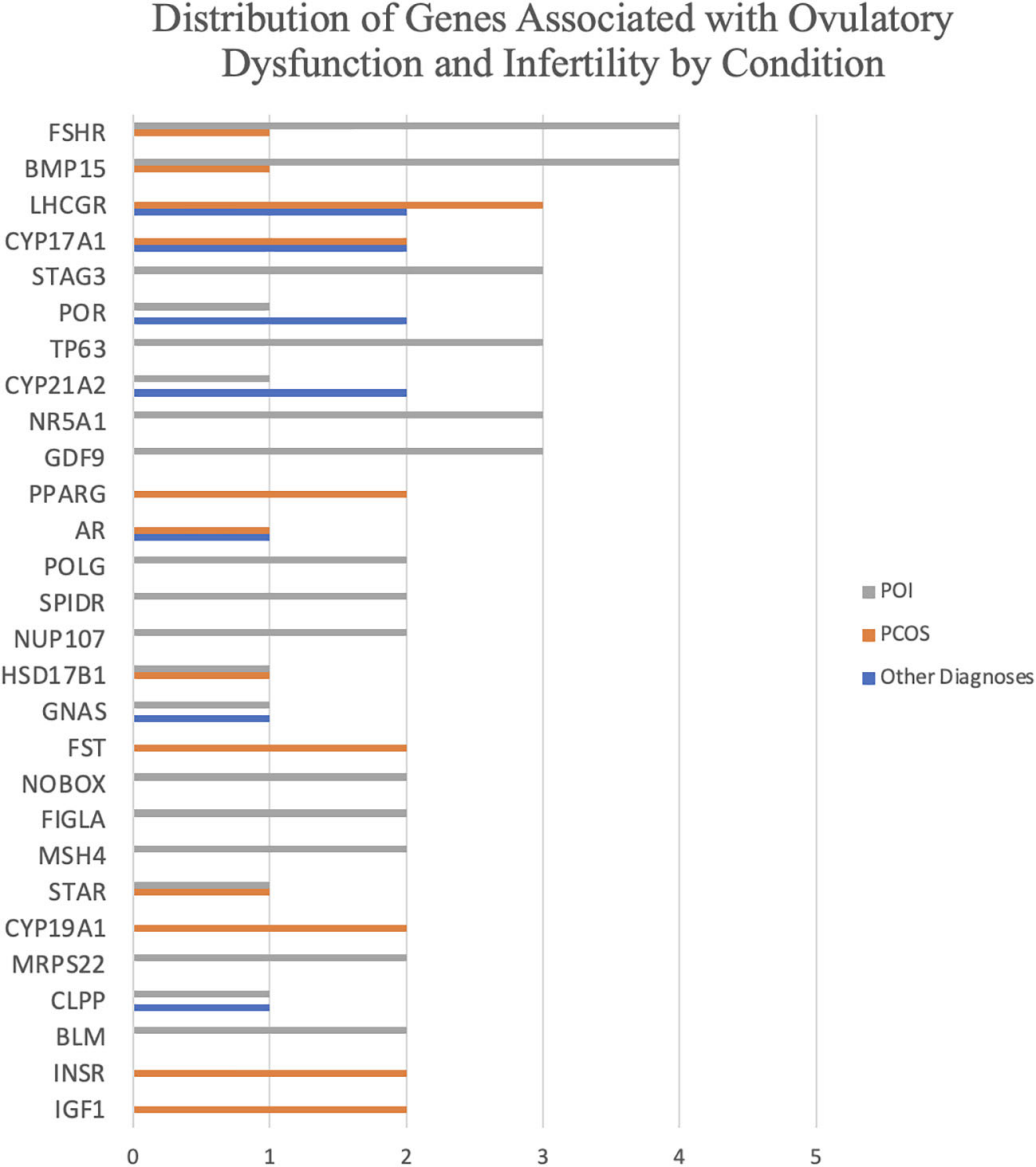
A bar graph showing the distribution of genes associated with ovulatory dysfunction and infertility sorted by gene name and stratified by condition type: PCOS, POI, or Other. Gene associations mentioned in one research article or less are excluded from this graph.

Out of 45 retrieved research articles (**Supplementary Table 1**) (22–66), there are 235 unique genes with variants or variable expression levels identified with some association with ODRI. While most gene variants or targets were uniquely identified in only one research article, some were observed at a higher frequency. From the list of 235 genes, 28 gene names were repeated in more than one research article (**Figure 2**), with 14 having been previously documented in the ClinVar database (20) to have pathogenic or likely pathogenic associations to ovulatory dysfunction, PCOS, or POI. Genes with associated variants mentioned in five articles include follicle-stimulating hormone receptor (*FSHR*), luteinizing hormone/choriogonadotropin receptor,\ (*LHCGR*), and bone morphogenic protein 15 (*BMP15*) (**Supplementary Table 2**). Genes identified in multiple research articles were considered higher confidence genes and were given increased scrutiny.

### Genes associated with polycystic ovary syndrome

**Figure 3:**
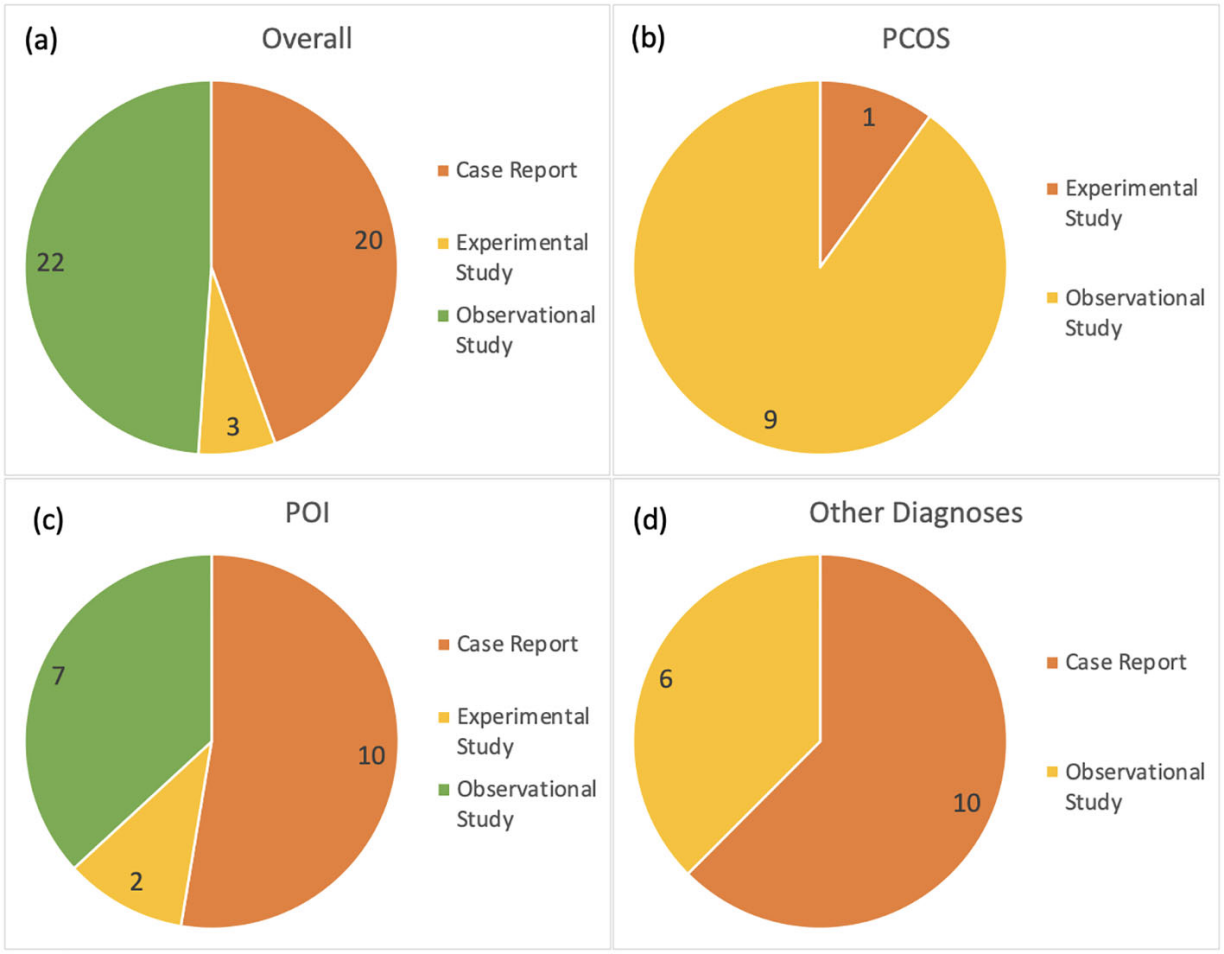
Distribution of research article types included in this scoping review; **(a)** including all subcategories, **(b)** articles about PCOS, **(c)** articles about POI, and **(d)** remaining articles referencing other categories of disease related to ovulatory dysfunction and infertility. The number of articles is included in the diagrams.

PCOS is identified in 10 of the reports included in this review. All articles report genetic variants in nuclear DNA. There were nine observational studies and one experimental study (**Figure 3**). There were 141 associated genes with PCOS identified in this review. Out of the PCOS-associated gene list, 12 were identified in more than one research article. The 12 higher confidence genes were *IGF1, INSR, CYP19A1, FST, PPARG, STAR, HSD17B1, BMP15, FSHR, AR, CYP17A1*, and *LHCGR.* Only four of these higher confidence genes have known pathogenic or likely pathogenic associations to ovulatory dysfunction according to the ClinVar database: *PPARG, BMP15, AR*, and *LHCGR.*

Eight of these articles identified gene variants involved with hormone modulation or synthesis. The earliest report by Urbanek et al. (1999) identified 37 gene loci in a differential gene expression analysis involved with the endocrine system, including androgen synthesis and function. Three papers have identified variants in the *LHCGR* gene for PCOS associations (**Figure 2**) (25, 42, 64). The gene is discussed at length by Atoum et al. (2022), discussing how different polymorphisms are associated with specific phenotypic qualities, including hirsutism, obesity, oligomenorrhea, luteinizing hormone, and follicle-stimulating hormone levels.

Ajmal et al. (2021) and Kaur et al. (2018) further identified specific variants in the *CYP19A1* and *CYP17A1* genes, respectively. The projected function of these genes is in estrogen synthesis, supporting the initial findings of Urbanek et al. (1999). Another gene involved with endocrine function is *INSR* for insulin sensitivity (59).

The remaining articles identified genes serving basic cellular functions. While one article directly identifies variants in genes involving cell proliferation and growth (35), others identify loss-of-function gene variants associated with cellular death and transcription regulation (22, 42, 60).

**Figure 4:**
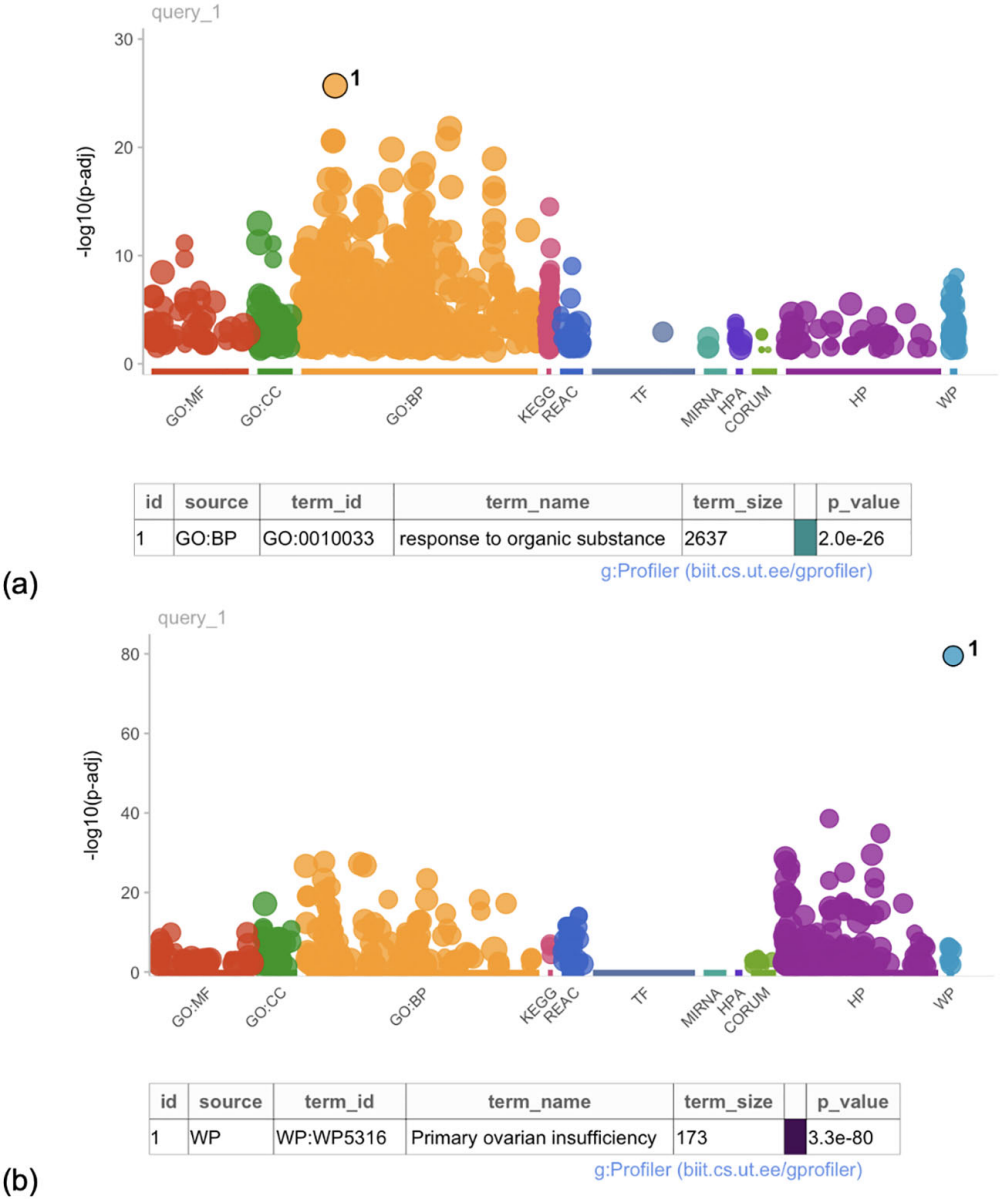
Manhattan plot of the gene ontology analysis for **(a)** PCOS-associated genes and **(b)** POI-associated genes. Each dot represents a gene ontology term associated with a gene name extracted from the literature. Gene ontology terms are considered enriched at a p-value < 1e-16.

The GO analysis revealed 16 gene functions that were highly enriched for the PCOS and infertility categories. All PCOS enriched terms were categorized in the biological process and included terms related to response to an organic substance (p = 2e-26, **Figure 4**), hormones, oxygen-containing compounds, as well as cell proliferation.

### Genes associated with premature ovarian insufficiency

A total of 19 reports identified genetic targets for POI in this review. Ten of these research articles are case reports. Two articles are experimental studies, and two are observational studies (**Figure 3**). The number of genes identified in this review associated with POI was 114. Out of these, 16 were identified in more than one research article. The 16 higher confidence genes were *BLM, MRPS22, GDF9, NR5A1, MSH4, FIGLA, NOBOX, NUP107, SPIDR, POLG, TP63, STAG3, CLPP, GNAS, CYP21A2*, and *POR.* Majority of the genes identified in this higher confidence gene list are documented in the ClinVar database as having a known association with ovulatory dysfunction of POI. There are nine potentially novel gene targets that are identified as potentially associated with POI in this review: *BLM, GDF9, NR5A1, STAR, HSD17B1, FSHR, CLPP, GNAS*, and *CYP21A2.*

One article identified a mitochondrial locus as a contributor to POI: *MRPS22* (49). Components of this gene are essential for mitochondrial DNA integrity, mitochondrial ribosomal composition, and ATP production (49, 67).

Gene variants in the POI literature suggest involvement in folliculogenesis and ovarian function. These variants are found in genes such as the *GDF9, NOBOX, NUP107, BMP15, NOTCH2*, and *NR5A1* genes (23, 28, 32, 37, 40, 41, 48, 50, 68). Hormone regulation and synthesis genes were presented by four authors (38, 50, 52, 68).

Variants in *FSHR*, a gene involved with the cAMP pathway (30) were documented in four POI-specific articles (**Figure 2**). Two case reports identified novel variants in the *FSHR* gene in separate populations. In Brazil, a novel homozygous missense mutation caused anovulation for two siblings (30). In Finland, a novel heterozygous mutation impacted the efficacy of the cAMP pathway and downstream caused anovulation (57).

Some gene variants were involved with DNA integrity, transcription, and translation. Yatsenko et al. (2022) reported a variety of variants in the *ZSWIM7* gene that are involved with DNA damage response. Franca et al. (2019) described two novel variants in the *STAG3* gene involved with genome expression and integrity (**Figure 3**). Caburet et al. (2014) identified a point mutation in this same gene leading to a premature stop codon, which impacts the development of a protein complex called cohesin. Cohesin plays a critical role in chromosome pairing during meiosis.

The GO term analysis revealed that the most highly enriched GO term across the list of gene names was premature ovarian insufficiency (*p* = 3.3e-80, **Figure 4**). In total, however, 43 enriched gene functions were identified as significant for the POI and infertility category. The enriched terms are related to biological process pathways and basic cellular functions. These terms included abnormal morphology of female reproductive physiology and genitalia, DNA damage response and repair, and recombination.

### Genes associated with other infertility-related diagnoses

Other diagnoses associated with ovulatory dysfunction and infertility range in type from symptomatic to asymptomatic conditions and account for 16 of the included reports. Ten of these reports are case report studies, and six are observational studies (**Figure 3**). The disorders include enzyme deficiencies, hormone deficiencies, and unexplained infertility.

The number of genes identified in this review associated with other infertility-related disorders was 21. Out of these, seven were identified in more than one research article. The seven higher confidence genes were *CLPP, GNAS, CYP21A2, POR, AR, CYP17A1*, and *LHCGR*. Upon review with the ClinVar database, three genes were identified with pathogenic or likely pathogenic associations to ovulatory dysfunction: *POR, AR*, and *LHCGR*. The remaining four genes should be investigated for their potential causal relationship with ODRI.

Lavery et al. (2008) presented a case report on a subject with cortisol reductase deficiency. Other cases of enzyme deficiencies appear to have variants in genes with functions related to hormones and or gonadal development (34, 46, 51).

Sahakitrungruang et al. (2009) and Papadakis et al. (2020) discuss genes involved with P450 oxidoreductase deficiency (PORD), highlighting the role of the *POR* and the *CYP21A2* genes. *CYP21A2* is also affected in Congenital Adrenal Hyperplasia, leading to secondary PORD. The function of these genes is critical for hormonal interaction, gonadal development, and folliculogenesis (34). The remaining articles referencing enzyme deficiencies affecting ovulation and fertility report on variants in genes from the same family of P450 enzymes critical for steroidogenesis: *CYP17A1, CYP19A1*, and *CYP21A2* (34, 46, 51).

For the research articles involving a diagnosis of hormone deficiency, the genes identified are *FSHB, FSHR, LHB, LHCGR*, and *GNRH1.* Upon review, the functions of these genes are related to ovulation, pubertal development, and hormonal regulation (43, 44, 53). The *LHCGR* gene is also identified as pathogenic in some cases of idiopathic infertility. Zhang et al. (2020) and Xu et al. (2023) specifically describe novel variants in the gene being correlated to empty follicle syndrome. All GO terms extracted from the gene list associated with “Other diagnoses” revealed no statistically significant findings.

## Discussion

This scoping review of the genetics of ODRI revealed a wide range of genetic variants, which are broadly associated with cell cycle components, inflammation, DNA integrity and endocrine functions (33, 46, 48, 55, 69). Through synthesizing the data from the articles included in this review, we highlighted differences in the gene functions based on the diagnosis categories: PCOS, POI, and “Other.” Performing a GO-term enrichment analysis further validated this observation: functions of genes with associations to each respective category yielded unique functional profiles.

### PCOS

Literature documenting gene associations with PCOS and infertility referenced variants in hormone-regulatory regions, consistent with the phenotypic points used in the current diagnostic criteria (22, 25, 46, 70). For example, specific polymorphisms in the *LHCGR* gene are associated with high levels of luteinizing hormone, sometimes measured as a key feature for PCOS diagnosis. It is theorized that an increase in follicle-stimulating hormone and luteinizing hormone levels explains the excess production of androgens typical of this syndrome (66, 71). Hyperinsulinemia is also theorized to increase the production of androgens in patients with PCOS, though this is through a process independent of the mechanism involving the *LHCGR* gene (39, 72). Knowledge of genetic underpinnings can help direct treatment options for these patients and help to better classify the subtypes of PCOS in a clinical setting.

### POI

Some of the genetic association patterns between PCOS and infertility are akin to those identified between POI and infertility in their function, though the individual genes identified are different. Similar functions observed in the two categories involve general cellular function and DNA repair (22, 31, 60, 61).

Specific variants in the *STAG3* gene in POI-related infertility are investigated in depth by Franca et al. (2019). Cohesin is critical for genome expression and integrity and contributes to the proper division of chromosomes during meiosis (54). Patients with complete anovulation carried pathogenic variants in *STAG3* in both reports (20).

The *FSHR* gene, involved with the cAMP pathway, is unique to the POI literature as defined by the constructs of this scoping review. This pathway is vital for pubertal development and ovulation (30). Other genetic associations with POI and infertility reveal a similar theme of pubertal development, folliculogenesis, and follicle maturation. These findings support the unique etiology of ovulatory dysfunction and infertility resulting from POI.

### Other diagnoses

The genetics of other diagnoses associated with ovulatory dysfunction and infertility were investigated, and 21 genes were identified. The GO-term enrichment analysis did not reveal specific patterns of functionality or characterization of the genes associated with other diagnoses extracted in this scoping review, which reveals an opportune area for future research and discovery.

Interestingly, the *LHCGR* gene is identified, as it was with the PCOS category. However, the implications of variants in the “other” category are unique. As a result of the variants described by Zhang et al. (2020) and Xu et al. (2023), patients with *LHCGR* mutations suffered empty follicle syndrome. Empty follicle syndrome is defined as an inability to retrieve oocytes at the time of surgical removal of follicles during an assisted reproductive treatment cycle (73). The effect that the *LHCGR* gene has on the LH receptor pathway may prevent downstream signaling, thus preventing changes in the collagen matrix and binding of oocytes, as well as ovulation (27, 33, 73).

On the topic of assisted reproductive technology, Papadakis et al. (2020) note that in patients with PORD, unassisted conception is not reported. This enzyme deficiency is considered an extreme form of CAH due to the severity of complications and presentation of symptoms, including genital ambiguity, amenorrhea, and infertility (29).

Given that the primary outcome of these conditions is ovulatory dysfunction and infertility, the diagnostic classification of disease based on phenotypic observations is suboptimal. Consider the case report presented by Lavery et al. (2008), a Cortisol Reductase Deficiency patient presented with a PCOS-like phenotype. After genetic analysis, the gene variants identified did not match known genetic profiles of PCOS patients. The authors illustrate how gene variants affecting the cortisol pathway, not androgens, can mimic the PCOS phenotype (74). Understanding the genetic underpinnings of ODRI conditions may help clinicians better tailor treatment strategies and opens an opportunity for new genetic technological applications in assisted reproductive technology (75).

### Limitations

The utilization of strict inclusion and exclusion criteria was both a limitation and a strength in this scoping review. The inclusion of only English language articles may have resulted in the omission of relevant findings, though necessary due to the article curation methodology employed. A single reviewer was responsible for the review of these articles, which may have contributed to article selection bias. This scoping review, however, employed several strategies to support the effectiveness and uniformity of article selection, such as article validation by a second and third reviewer and the use of strict criteria.

By not limiting the timeframe, genetic testing types varied significantly from Sanger sequencing to whole exome sequencing. All research articles that were identified at initial article collection contained significant clinical genetic findings, suggesting that there is inherent publication bias around the genetics of ODRI. No articles identified revealed evidence that ODRI does not have a genetic cause or association.

Although the choice to omit articles due to chromosomal aberration or epigenetic modulations may have impacted the volume of articles that could have been included, it was done so with intention to focus on single nucleotide polymorphisms and variants. This contributes to ODRI as this is an area that is poorly understood.

Many of the articles included in this review were case reports with few or less subjects involved in the study. Although the small sample sizes in these case studies impact the statistical power and reduce the generalizability of the respective papers, the inclusion of all articles that met criteria was imperative to generating a comprehensive list of ODRI genes. Adding a quality scoring component was not performed to maximize the breadth of this scoping review and to identify a broad spectrum of potential gene targets for ODRI for future use.

A comprehensive review was not performed on all 235 genes, rather this was only performed on genes identified in multiple research articles to focus on genes with a higher confidence. This may have resulted in an incomplete analysis of rare pathogenic genetic variants within the analysis that was performed herein. However, the list of 235 genes was successfully compiled and is available for future research and development efforts.

Though there are limitations, this scoping review recovered germane and interesting data from the two largest literature databases. We looked at primary research articles from around the world, which assures diversity. While a large proportion of articles were case studies, including them allowed for a wider view of an area of research that is poorly studied. Additionally, a systematic approach was employed for this scoping review. Utilization of a systematic approach in combination with a quality assurance analysis increases our confidence in the findings extracted from the literature included in this review.

### Future directions

This research presents a large and comprehensive list of genes related to ODRI, which have potential applications ranging from research to clinical utility. In research, a list of vetted genes is particularly useful for targeted bioinformatics analyses on large datasets and can be used to discover potentially pathogenic mechanisms related to ODRI (76). This could contribute to understanding the pathogenic mechanisms underlying other types of infertility and discovering novel roles of the candidate genes. Research that helps to identify genetic underpinnings of ODRI could contribute to future clinical strategies through the development or improvement of tools used in infertility treatment, such as biomarker panels and next generation sequencing panels (77, 78).

## Conclusion

In this scoping review, we have highlighted the importance of an ongoing investigation on the genetics of ODRI in the literature. We have identified 235 genes related to ODRI in the literature specific to humans, with 28 genes demonstrating a higher potential relationship to ODRI. Our findings provide a comprehensive list of genes available for use in future research and clinical applications.

The list of ODRI genes can be used for targeted gene panel development in assisted reproductive technology to improve clinical testing and diagnosis. More specifically, the use of genetic profiling for patients with ovulatory dysfunction and infertility has the potential to improve patient experience and develop individualized treatments in the future. Our findings support the idea that utilizing genetic testing in infertility treatment may also allow for better classification and treatment of infertility disorders (5, 12, 14, 16), particularly in the realm of ODRI.

## Data Availability

The original contributions presented in the study are included in the article/**Supplementary Material**. Further inquiries can be directed to the corresponding author.

## Supplementary material

Supplementary Table 1 Data extraction table containing journal article demographics and categorical data. See attached File “OvDys_DataExtraction_DiPietro.xlsx”.

Supplementary Table 2 A list of the 28 gene names with associated variants included in more than one research article with associations to ovulatory dysfunction and infertility where “N” is equal to the number of research articles that mention variants in that specific gene. Genes with a (*) indicate at least one likely pathogenic or pathogenic variant related to ovarian dysfunction, or the respective conditions is present in the ClinVar database.
