# Supplementary material for: Genetics of ovulatory dysfunction and infertility: a scoping review and gene ontology analysis
Source: Front Endocrinol (Lausanne). 2025 Jun 4;16:1458711. doi: 10.3389/fendo.2025.1458711 (PMC12173923; doi:10.3389/fendo.2025.1458711)
Supplement: Supplementary Table 2 — A list of the 28 gene names with associated variants included in more than one research article with associations to ovulatory dysfunction and infertility where “N” is equal to the number of research articles that mention variants in that specific gene. Genes with a (*) indicate at least one likely pathogenic or pathogenic variant related to ovarian dysfunction, or the respective conditions is present in the ClinVar database. [file Table2.docx]

Supplementary Table 2: A list of the 28 gene names with associated variants included in more than one research article with associations to ovulatory dysfunction and infertility where “N” is equal to the number of research articles that mention variants in that specific gene. Genes with a (*) indicate at least one likely pathogenic or pathogenic variant related to ovarian dysfunction, or the respective conditions is present in the ClinVar database.

| Gene Name | Ensembl  ID | Location | Number of Research Articles Referencing Gene of Interest | | | |
| --- | --- | --- | --- | --- | --- | --- |
|  |  |  | **PCOS**  **(N)** | **POI**  **(N)** | **Other (N)** | **Grand Total (N)** |
| *insulin like growth factor 1*  *IGF1* | ENSG00000017427 | Chromosome 12: 102,395,874-102,481,744 reverse strand. | 2 |  |  | 2 |
| *insulin receptor*  *INSR* | ENSG00000171105 | Chromosome 19: 7,112,255-7,294,414 reverse strand. | 2 |  |  | 2 |
| *cytochrome P450 family 19 subfamily A member 1*  *CYP19A1* | ENSG00000137869 | Chromosome 15: 51,208,057-51,338,601 reverse strand. | 2 |  |  | 2 |
| *follistatin*  *FST* | ENSG00000134363 | Chromosome 5: 53,480,626-53,487,134 forward strand. | 2 |  |  | 2 |
| *peroxisome proliferator activated receptor gamma*  *PPARG* | ENSG00000132170 | Chromosome 3: 12,287,368-12,434,356 forward strand. | 2 |  |  | 2* |
| *BLM RecQ like helicase*  *BLM* | ENSG00000197299 | Chromosome 15: 90,717,346-90,816,166 forward strand. |  | 2 |  | 2 |
| *mitochondrial ribosomal protein S22*  *MRPS22* | ENSG00000175110 | Chromosome 3: 139,005,806-139,360,497 forward strand. |  | 2 |  | 2* |
| *growth differentiation factor 9*  *GDF9* | ENSG00000164404 | Chromosome 5: 132,861,181-132,866,884 reverse strand. |  | 3 |  | 3 |
| *nuclear receptor subfamily 5 group A member 1*  *NR5A1* | ENSG00000136931 | Chromosome 9: 124,481,236-124,507,420 reverse strand. |  | 3 |  | 3 |
| *mutS homolog 4*  *MSH4* | ENSG00000057468 | Chromosome 1: 75,796,882-75,913,242 forward strand. |  | 2 |  | 2* |
| *folliculogenesis specific bHLH transcription factor*  *FIGLA* | ENSG00000183733 | Chromosome 2: 70,777,310-70,790,643 reverse strand. |  | 2 |  | 2* |
| *NOBOX oogenesis homeobox*  *NOBOX* | ENSG00000106410 | Chromosome 7: 144,397,240-144,410,227 reverse strand. |  | 2 |  | 2* |
| *nucleoporin 107*  *NUP107* | ENSG00000111581 | Chromosome 12: 68,686,951-68,745,809 forward strand. |  | 2 |  | 2* |
| *scaffold protein involved in DNA repair*  *SPIDR* | ENSG00000164808 | Chromosome 8: 47,260,878-47,736,306 forward strand. |  | 2 |  | 2* |
| *DNA polymerase gamma, catalytic subunit*  *POLG* | ENSG00000140521 | Chromosome 15: 89,305,198-89,334,861 reverse strand. |  | 2 |  | 2* |
| *tumor protein p63*  *TP63* | ENSG00000073282 | Chromosome 3: 189,631,389-189,897,276 forward strand. |  | 3 |  | 3* |
| *STAG3 cohesin complex component*  *STAG3* | ENSG00000066923 | Chromosome 7: 100,177,563-100,214,387 forward strand. |  | 3 |  | 3* |
| *steroidogenic acute regulatory protein*  *STAR* | ENSG00000147465 | Chromosome 8: 38,142,700-38,150,992 reverse strand. | 1 | 1 |  | 2 |
| *hydroxysteroid 17-beta dehydrogenase 1*  *HSD17B1* | ENSG00000108786 | Chromosome 17: 42,552,922-42,555,214 forward strand. | 1 | 1 |  | 2 |
| *bone morphogenetic protein 15*  *BMP15* | ENSG00000130385 | Chromosome X: 50,910,735-50,916,641 forward strand. | 1 | 4 |  | 5* |
| *follicle stimulating hormone receptor*  *FSHR* | ENSG00000170820 | Chromosome 2: 48,962,157-49,154,527 reverse strand. | 1 | 4 |  | 5 |
| *caseinolytic mitochondrial matrix peptidase proteolytic subunit*  *CLPP* | ENSG00000125656 | Chromosome 19: 6,361,531-6,370,242 forward strand. |  | 1 | 1 | 2 |
| *GNAS complex locus*  *GNAS* | ENSG00000087460 | Chromosome 20: 58,839,718-58,911,192 forward strand. |  | 1 | 1 | 2 |
| *cytochrome P450 family 21 subfamily A member 2*  *CYP21A2* | ENSG00000198457 | Scaffold HSCHR6_MHC_SSTO_CTG1: 3,339,488-3,342,857 forward strand. |  | 1 | 2 | 3 |
| *cytochrome p450 oxidoreductase*  *POR* | ENSG00000127948 | Chromosome 7: 75,899,200-75,986,855 forward strand. |  | 1 | 2 | 3* |
| *androgen receptor*  *AR* | ENSG00000169083 | Chromosome X: 67,544,021-67,730,619 forward strand. | 1 |  | 1 | 2* |
| *cytochrome P450 family 17 subfamily A member 1*  *CYP17A1* | ENSG00000148795 | Chromosome 10: 102,830,531-102,837,472 reverse strand. | 2 |  | 2 | 4 |
| *luteinizing hormone/ choriogonadotropin receptor*  *LHCGR* | ENSG00000138039 | Chromosome 2: 48,686,774-48,755,730 reverse strand. | 3 |  | 2 | 5* |
